# Temperature-Dependent Asymmetry of Anisotropic Magnetoresistance in Silicon *p-n* Junctions

**DOI:** 10.1038/srep11096

**Published:** 2015-09-01

**Authors:** D. Z. Yang, T. Wang, W. B. Sui, M. S. Si, D. W. Guo, Z. Shi, F. C. Wang, D. S. Xue

**Affiliations:** 1 Key Laboratory for Magnetism and Magnetic materials of Ministry of Education, Lanzhou University, Lanzhou 730000, China; 2 The Department of physics, Tongji University, Shanghai 200092, China

## Abstract

We report a large but asymmetric magnetoresistance in silicon *p*-*n* junctions, which contrasts with the fact of magnetoresistance being symmetric in magnetic metals and semiconductors. With temperature decreasing from 293 K to 100 K, the magnetoresistance sharply increases from 50% to 150% under a magnetic field of 2 T. At the same time, an asymmetric magnetoresistance, which manifests itself as a magnetoresistance voltage offset with respect to the sign of magnetic field, occurs and linearly increases with magnetoresistance. More interestingly, in contrast with other materials, the lineshape of anisotropic magnetoresistance in silicon *p*-*n* junctions significantly depends on temperature. As temperature decreases from 293 K to 100 K, the width of peak shrinks from 90° to 70°. We ascribe these novel magnetoresistance to the asymmetric geometry of the space charge region in p-n junction induced by the magnetic field. In the vicinity of the space charge region the current paths are deflected, contributing the Hall field to the asymmetric magnetoresistance. Therefore, the observed temperature-dependent asymmetry of magnetoresistance is proved to be a direct consequence of the spatial configuration evolution of space charge region with temperature.

Modern information technology is based on reading, writing and storing data encoded as magnetic bits^1^. Thus, materials featuring large magnetoresistance (MR) have been widely used in magnetic sensors^2^, hard drives^3^ and magnetic memory^4^. Usually, magnetic materials are expected to possess a large MR, such as the anisotropic magnetoresistance (AMR) in ferromagnetic metals^5^, the giant magnetoresistance (GMR) in multilayers^3^,^6^, the tunneling magnetoresistance (TMR) in tunnel junctions^7^, and the colossal magnetoresistance (CMR) in perovskites^4^,^8^. Although different mechanisms are proposed to understand the MR effects in magnetic materials, all these MR effects share the common symmetry with respect to external magnetic field (*H*), namely MR (*H*) = MR (*−H*).

For conventional nonmagnetic materials, ordinary MR is relatively weak compared to those reported in magnetic materials, but the common MR symmetry still holds. Recently, this situation is changed in some novel nonmagnetic materials such as doped silver chalcogenide^9^,^10^, indium antimonide^11^, niobium selenium^12^, iron diantinomide^13^, gallium arsenide^14^, germanium^15^ and silicon^16^,^17^,^18^,^19^,^20^,^21^,^22^,^23^. All of them have extremely large and non-saturating MR of up to several thousand per cent in a magnetic field of several teslas. More intriguingly, the common MR symmetry, which widely holds in magnetic and conventional nonmagnetic materials, seems to be broken among these novel materials. For example, an obvious MR offset with respect to the sign of magnetic field was reported by Sinchenko *et al*.^12^ and Delmo *et al*.^17^, reflecting an asymmetric MR as MR (*H*) ≠ MR (−*H*). Although large and non-saturating MR has been widely studied by considering as inhomogeneous conductors^24^,^25^,^26^,^27^,^28^ or quantum theory^29^,^30^, a study of asymmetry MR in nonmagnetic materials is still lacking.

In this work, we report an observation of two asymmetric MR features in the silicon p-n junctions. With temperature decreasing from 293 K to 100 K, an MR voltage offset with respect to the sign of magnetic field occurs and linearly increases with MR. By systematically measuring the angular dependence of MR, we further found the lineshape of AMR is temperature-dependent. These are both contrast with the conventional materials. We ascribe these newly discovered asymmetric MR of p-n junctions to the asymmetric geometry of the space charge region induced by the magnetic field, in which the current path deflection contributes the Hall field to the asymmetry of MR.

## Results and Discussions

### Fabrication and measurement of p-n junction devices

**Figure 1:**
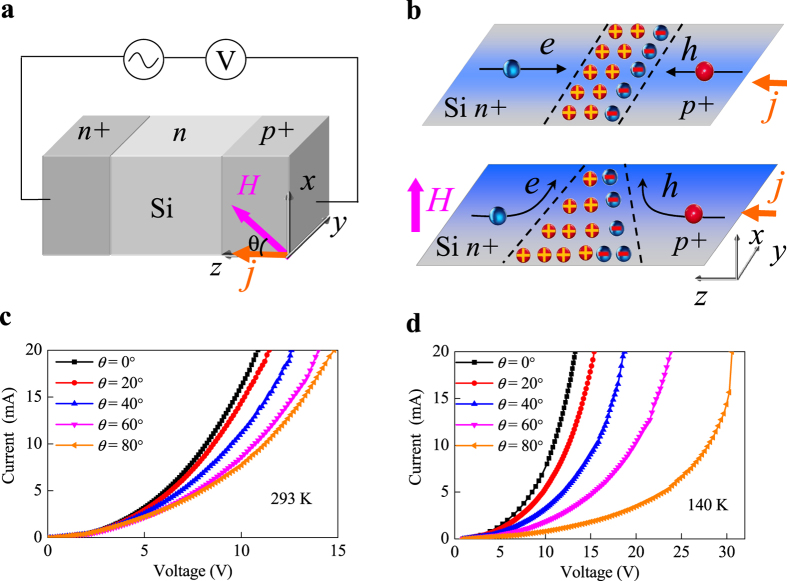
The transport characteristics of the p-n junctions modulated by the magnetic field. (**a**) The p-n junction device structure and its measurement (**b**) Schematic illustration of the origin of the MR effect in p-n junctions due to the change of the space charge region tuned by magnetic field. Without the magnetic field a uniform distribution in space-charge region is formed, while under magnetic field a trapezoidal distribution of space charger region is formed due to the Lorentz force and the current trajectory are deflected toward the lower barriers. The corresponding *I-V* curves of the p-n junctions under the magnetic field *H* = 2 T with angle *θ* from 0° to 90° at 293 K (**c**) and 140 K (**d**), demonstrating the significant temperature-dependent anisotropic MR effect.

The schematic illustration of the p-n junction device structure as well as the measurement diagram is shown in Fig. 1a. The vertical geometry of Si(p+)/Si(n)/Si(n+) is chosen to form a wide space charge region. At room temperature the carrier densities of Si(p+), Si(n), and Si(n+) were 2.0 × 10^14^  cm^−3^, 1.0 × 10^12^ cm^−3^ and 1.0 × 10^15^  cm^−3^, respectively. The angular dependence of MR was measured by rotating the sample holder under a fixed magnetic field *H* = 2 T and a current *I* = 20 mA. The orientation of *H* was defined as *θ*, which is the angle between the *H* and *z* axes. During the whole measurement the magnetic field is rotated in the *x–z* plane. Here the MR ratio is defined as (R(H)−R(0))/R(0) × 100%.

### Magnetic field modulated transport characteristics of p-n junctions

Figure 1b shows the schematic of the space-charge region in bipolar p-n junction that gives rise to the interesting properties electrically and magnetically^31^. Without the magnetic field the space charge region is uniform. However, by tuning the width of space charge region by external electric field, the resistance of p-n junction can change in the range of several orders of amplitude. This is also known as the rectifying effect in diode. Similar to the rectifying effect under electric field, the change of space charge region under the magnetic field can also be expected to remarkably tune the resistance of p-n junction. As shown in Fig. 1b, under the magnetic field the carriers in n-type and p-type region are deflected by the Lorentz force and accumulated at the edges of the sample. As a result, a trapezoidal distribution of the space charge region is formed and caused a large MR effect.

To confirm such scenario for MR effect of p-n junction, the current (*I*) - voltage (*V*) characteristics of p-n junction device were measured for various magnetic field orientations with temperatures from 100 K to 293 K. During all the measurements the amplitude of magnetic field was fixed to be 2 T. Figure 1c,d show the typical transport characteristics of p-n junction at 293 K and 140 K, respectively. All the *I*-*V* curves present the obvious nonlinear behaviors due to the intrinsic space charge region. At *T* = 293 K for *θ* = 0°, that is the magnetic field is parallel with the current, the junction voltage is about 10 V at *I* = 20 mA, as shown in Fig. 1c. However, with the *θ* increasing from 0° to 80°, the junction voltage gradually increases, indicating a significant angular dependence of MR effect. For *θ* = 80°, at the same current *I* = 20 mA, the junction voltage is shifted forward to 15 V, thus the corresponding MR is up to 50%, which is one order of magnitude larger than that of magnetic materials (e.g. Permalloy). This large anisotropic MR effect can be explained by the angular dependence of Lorentz force that affects the spatial distribution of space charge region^32^. With the temperature going down, the anisotropic MR effect sharply increases. As shown in Fig. 1d, at 140 K with *θ* increasing from 0° to 80°, the junction voltage increases from ~12 V to 30 V for the same current level *I* = 20 mA, indicating the MR close to 150% . However, carefully comparing Fig. 1c with Fig. 1d, one can find that for the fixed current level the junction voltage change rates of *I-V* curves with *θ* step 20° are different. At 293 K for specified current (e.g. *I* = 20 mA), the maximum change rate of junction voltage occurs at *θ* between 40° and 60°, while at 140 K the maximum rate change of junction voltage occurs at *θ* between 60° and 80°. This means that our observed angular dependence of MR depends on temperature.

### Temperature-dependent AMR in p-n junctions

**Figure 2:**
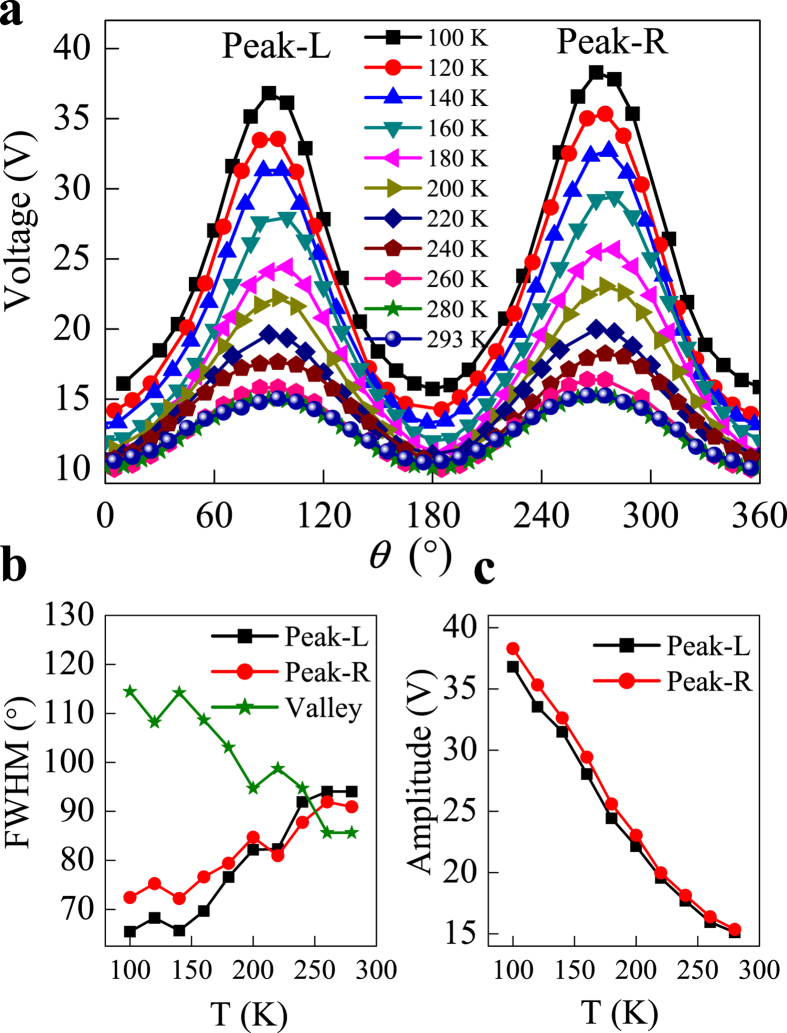
Temperature-dependent asymmetry of anisotropic MR of p-n junctions. (**a**) The angular dependent of measured MR curves at specified current *I* = 20 mA and magnetic field *H* = 2 T. The anisotropic MR curves are gradually shown asymmetric both peak amplitude and the peak shape (FWHM) with decreasing temperature. (**b**) The temperature dependence of the FWHM of anisotropic MR curve at *θ* = 90° (Peak-L), 270° (Peak-R) and 180° (Valley). (**c**) The temperature dependence of the amplitude of anisotropic MR curve at *θ* = 90° (Peak-L) and 270° (Peak-R).

In order to study the temperature-dependent AMR, we measured the angular dependence of MR effect in p-n junction from 100 K to 293 K, as shown in Fig. 2a. One can easily find that for all temperatures, MR of p-n junction presents an obviously angular dependence. At room temperature the angular dependence of the MR is perfectly symmetric which is almost proportional to sin^2^(*θ*)^32^, while at low temperature the angular dependence of the MR presents two asymmetric features. One is the temperature-dependent lineshape of AMR. As shown in Fig. 2a, the peak (valley) positions are always at *θ* = 90° (0°) and 270° (180°), where magnetic field is perpendicular (parallel) with the current. However, with the temperature decreasing from 293 K to 100 K, the width of peak gradually shrinks from 90° to 70°. The detailed temperature dependence of the full width at half maximum (FWHM) at the peak and valley are also shown in Fig. 2b. Remarkably, this temperature dependence is in stark contrast to magnetic materials, in which lineshape of AMR is independent of temperature. Moreover, just this temperature dependence causes the different change rates with magnetic field orientations, as we discussed in Fig. 1c,d. The other one is the asymmetry of MR with respect to the magnetic field or MR(H) ≠ MR(−H). Comparing with the amplitude of the two peaks at *θ* = 90° and 270° in Fig. 2a, one can find a voltage offset with respect to the sign of the magnetic field. In Fig. 2c, we further draw the two peak amplitudes at *θ* = 90° and 270° as a function of the temperature. It is seen that with the temperature decreasing the voltage offset between the amplitude of two peaks gradually increases. Again, this asymmetry contrasts with the common symmetry of MR in magnetic materials.

**Figure 3:**
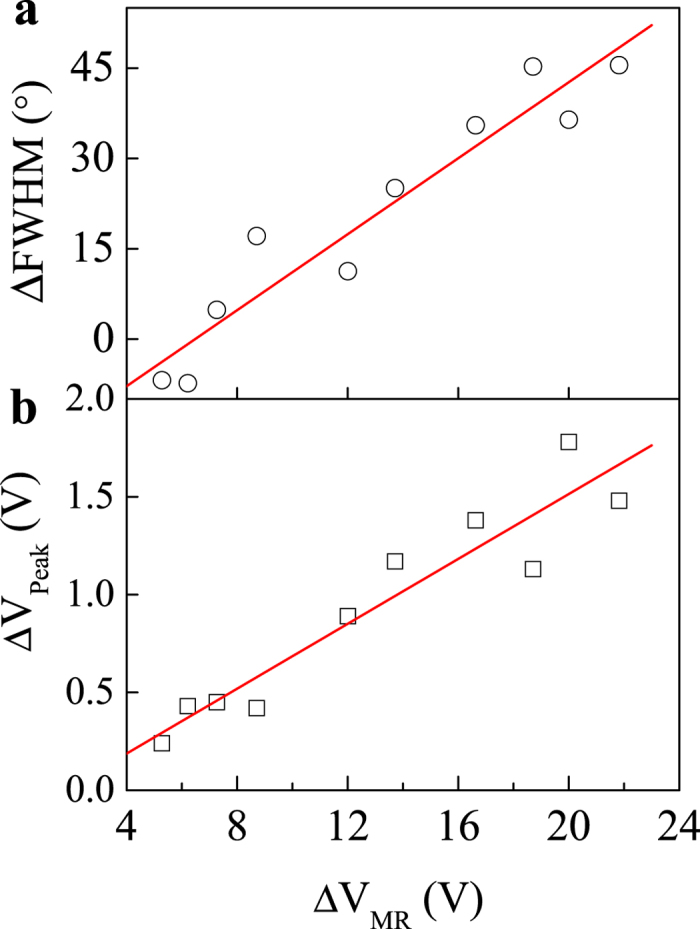
The linear relationship between the asymmtric features and the increment of MR voltage. (**a**) The FWHM offsets for peak and valley increases linearly with the increment of MR voltage for *H* = 0 and 2 T. (**b**) The peak amplitude offsets for *θ *= 90 and 270*°* increases linearly with the increment of MR voltage for *H* = 0 and 2 T.

In Fig. 3, we show the correlation of the two asymmetric behaviors with the increase MR voltage induced by magnetic field. Here we introduce two parameters Δ*θ*_FWHM_ and Δ*V*_Peak_ to characterize these two types of asymmetric effects. The Δ*θ*_FWHM_ = *θ*_FWHM_(180°) − *θ*_FWHM_(90°), which is defined as the angle difference of FWHM between the valley (*θ* = 180°) and peak (*θ* = 90°) in Fig. 2a. The Δ*V*_Peak_ = *V*_Peak-L_(90°) − *V*_Peak-R_(90°), which is defined as the voltage offset between the Peak-L (*θ* = 90°) and Peak-R (*θ* = 270°) in Fig. 2a. Interestingly, one can see that the two asymmetry parameters Δ*θ*_FWHM_ and ΔV_Peak_ both linearly increase with the increasing MR effect, as shown in Fig. 3a,b, respectively. By linearly fitting the data, the offsets are −0.14 V and −20.4° for ΔV_Peak_ and Δ*θ*_FWHM_ respectively, which are close to zeros. The proportional behaviors indicate that the asymmetry MR effects stems from the same mechanism of the large MR effect in p-n junctions.

### Explanation of temperature dependence of AMR in p-n junctions

**Figure 4:**
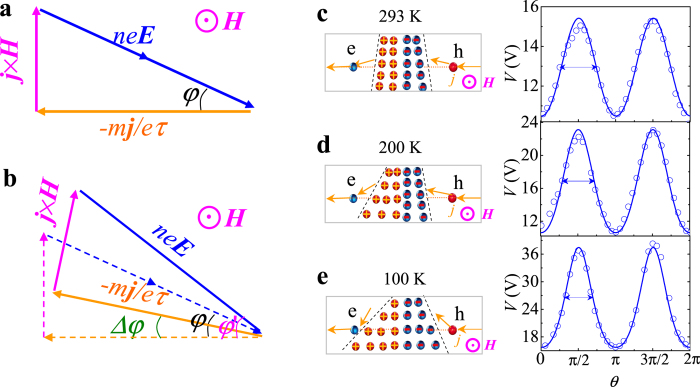
Interpretation of the observed asymmetric anisotropic MR due to the current deflection. (**a**) The vector diagram presented the forces equilibrium acting on the carriers under the magnetic field. For the conventional metal and semiconductor, the current trajectory keeps constant due to the adjustment of the electric field. (**b**) For the p-n junctions, the current trajectory is deflected due to the geometry of space charge region, as shown in the solid lines. (**c**–**e**) The schematic illustration of the current deflection in p-n junction and corresponding typical anisotropic MR curves for various temperature. With temperature decreasing, the space charge region is more efficiently affected by the magnetic field, thus the FWHM of AMR obviously decreases (marked as arrow) and the MR ratio significantly increases. The solid lines are fitted by the equation (1).

In order to explain our results, in Fig. 4a we draw a vector diagram with the solid line to present the forces equilibrium acting on the carriers in conventional metals and semiconductors. One sees that a Hall electric field arises to compensate the Lorentz force, but the component of electric field parallel to current is unchanged, indicating longitudinal resistivity should be independent on the magnetic field. However, if the current trajectory is deflected *Δφ* and the Hall angle is changed from *φ* to *φ*’, as shown in Fig. 4b, then the new equilibrium is formed. Interestingly, in this case the longitudinal resistivity *ρ*’ is changed as


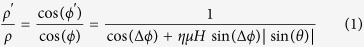


where we use *φ* = *φ*’ − *Δφ* to extend the cos(*φ*) and replace *φ*’ with Hall angle equation tg(*φ*’) = *ημ*Η|sin(*θ*)|, *η* is the MR coefficient, *μ* is the carrier mobility, *Η*|sin(*θ*)| is the component of the magnetic field perpendicular to the current plane and the absolute function indicates that the longitudinal resistivity is the same when the sign of magnetic field is changed. In general, due to the compensation of the Hall field, the current trajectory deflection shown in Fig. 4b can not occur in conventional materials. However, for the p-n junctions the space charge region can be changed by the magnetic field, thus it naturally induces that the current trajectory deflects toward the lower barrier, as shown in Fig. 1b. As a result, when the current deflection in p and n region are not equal, it causes the average current trajectory deflection with angle *Δφ*. As *Δφ* varies with the configuration of space charge region under magnetic field, the lineshape of anisotropic MR curves is correspondingly changed. From Fig. 4c–e, we further demonstrate the typical angular dependence of MR behaviors via the change of *Δφ*. At 293 K shown in Fig. 4c the space charge region of p-n junctions is symmetric, *Δφ* is almost zero and the MR curve closely follows sin^2^(*θ*). However, with the temperature decreasing as shown from Fig. 4c–e, the configuration of space charge region under magnetic field gradually varies with the increase of the carrier mobility. As a consequence, the geometry of the space charge region in p-n junctions become asymmetric, thus causes the change of the lineshape of AMR. We use the arrows to mark the FWHM of AMR peak. One can find that the FWHM of AMR obviously decreases, at the same time the MR ratio significantly increases with temperature decreasing. Because the MR asymmetry and MR ratio are both affected by the current deflection, it is also qualitatively agreement with the result that the linear relationship between MR asymmetry and MR ratio in Fig. 3. The picture also indicates that the MR ratio in p-n junctions can be further improved by designing a proper asymmetric space-charge region due to the current deflection.

**Figure 5:**
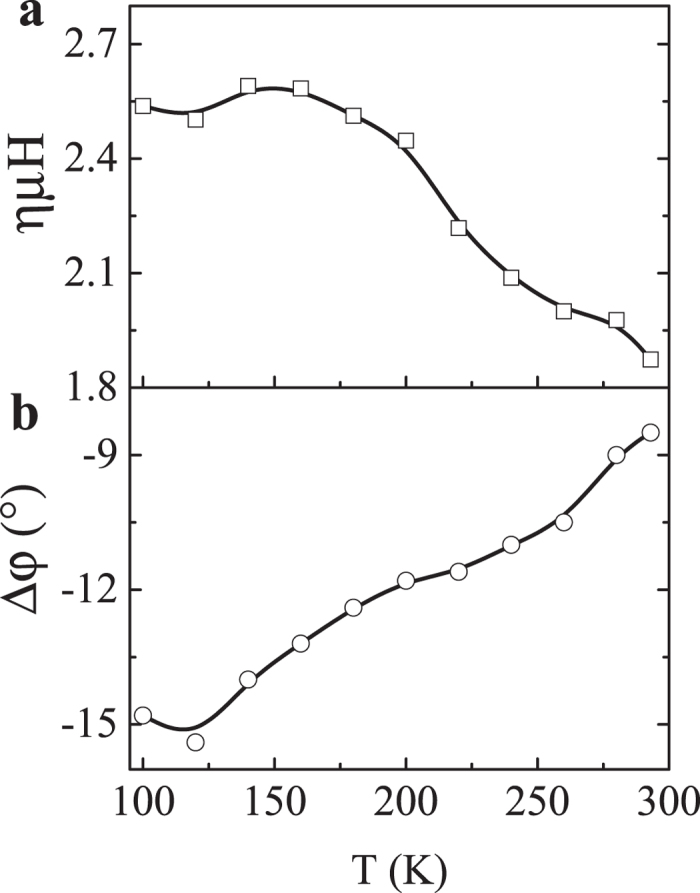
Fitted current deflection parameters. According to the equation (1), the parameters (**a**) *ημH* and (**b**) *Δφ* are fitted as a function of temperature. The lines are guided to eye.

We fitted all the AMR data with various temperatures well with equation (1). The two corresponding fitting parameters *ημH* and *Δφ* are obtained and shown in Fig. 5a,b, respectively. For Si, the mobility *μ* is about 1000 cm^2^/V·s at room temperature, *η* can be obtained to be ~9 under the magnetic field H of 2 T. We ascribe the large MR coefficient *η* to the amplification effect due to the space charge region change under magnetic field, as we discussed in Fig. 1b. With the temperature decreasing, the space charge region is more efficiently affected by the magnetic field (See Fig. S1 in the supplementary file). As a result, *ημH* and |*Δφ*| increase as shown in Fig. 5a,b. The fitted *Δφ* is negative, indicating that under the magnetic field the deflection of electron in *n* region is larger than the deflection of hole in p region. This is consistent with our explanation, because in silicon the electron mobility (1350 cm^2^/V·s) is much larger than that of the hole mobility (480 cm^2^/V·s).

On the other hand, the broken symmetry MR (*H*) ≠ MR (−*H*) usually indicates the violation of the time reversal invariance. It has been also observed in some cases of ferromagnetic material^33^,^34^,^35^,^36^, such as mesoscopic spin glasses^33^,^34^, in which the frozen scattering potential of magnetic ions in mesoscopic regimes breaks the time reversal. However, this is not true in our case due to lack of magnetic moment. We ascribe the origin of this asymmetry MR effect to the current deflection, which causes an additional Hall voltage at the boundary. When magnetic field orientation is rotated from *θ* to −*θ* in *x*–*z* plane, the magnetic field component along *x* axis is changed from *H*_*x*_ to −*H*_*x*_ and current along *y* axis is changed from *I*_*y*_ to −*I*_*y*_, but the Hall voltage along *z* axis current is still unchanged. This unchanged boundary Hall voltage contributes to the spatially inhomogeneous distribution of electric field, thus giving rise to an asymmetric MR. Remarkably, in some other nonmagnetic material with large MR effect this broken symmetry is also reported. For In/Intrinsic-Si/In at 300 K with magnetic field 3 T, the MR ratio is about 48% (voltage bias 80 V) and 120% (voltage bias 150 V), and the MR ratio caused by the asymmetric offset is up to 5% and 20%, respectively^17^. For NbSe_3_ single crystal at 4.2 K with magnetic field 8 T, the MR ratio is about 800%, but the MR ratio caused by asymmetric offset can even reach up to 600%^12^. Interestingly, in these materials, it is also shown that the asymmetry MR behavior is more significant with the larger MR ratios, which coincides with our experiment results.

## Conclusion

Hence, we conclude that we observed two asymmetric features both in amplitude and lineshape of the AMR curves in p-n junctions, which relied on the temperature. With the temperature decreasing, these two asymmetric features significantly increase and are further found to be proportional to MR. These newly discovered effects can be satisfactorily explained by the current path deflection due to the asymmetric geometry in space charge region under the external magnetic field. Although our discussion focuses on the configuration of the space charge region in p-n junctions, the mechanism itself does not rely on the space charge region and should also be helpful to explain asymmetry MR in nonmagnetic materials with large spatial inhomogeneity.

## Methods

The p-n junction devices were fabricated by the MEMS (Micro Electro Mechanical Systems). The wafers were lightly doped with 10^12^ atom/cm^3^ n-type dopant to achieve a good surface resistivity higher than 2000 Ω·cm. An oxidation film with a thickness of 6000 Å was grown on the wafers in the oxidation furnace at 1030 °C for 4 hours. After that, the micro-strip patterns were transferred to the wafers by a lithography machine. Then the wafers were further treated with a boron implantation (40 keV, 2 × 10^14^ atom/cm^3^) at top surface and a phosphorus implantation (60 keV, 1 × 10^15^ atom/cm^3^) at bottom surface by a medium-energy ion implanter. Finally, the Cu electrodes at the top and bottom were sputtered separately with the high vacuum 3 × 10^−5^ Pa. According to the simulation, the depths of ions implantation for (p+) and (n+) were about 144 nm and 82 nm, respectively. The junction size is about 6 mm × 3 mm × 0.5 mm.

## Additional Information

**How to cite this article**: Yang, D. Z. *et al*. Temperature-Dependent Asymmetry of Anisotropic Magnetoresistance in Silicon *p-n* Junctions. *Sci. Rep*. **5**, 11096; doi: 10.1038/srep11096 (2015).

## Supplementary Material

Supplementary Information

## References

[b1] Chappert C., Fert A. & Van Dau F. N. The emergence of spin electronics in data storage. Nature Matter. 6, 813–823 (2007). 10.1038/nmat2024 17972936

[b2] Lenz J. E. A review of magnetic sensors. Proc. IEEE 78, 973–989 (1990).

[b3] Daughton J. GMR applications. J. Magn. Magn. Mater. 192, 334–342 (1999).

[b4] Moritomo Y., Asamitsu A., Kuwahara H. & Tokura Y. Giant magnetoresistance of manganese oxides with a layered perovskite structure. Nature 380, 141–144 (1996).

[b5] Mcguire T. R. & Potter R. I. Anisotropic magnetoresistance in ferromagnetic 3d alloys. IEEE Trans. Mag. 11, 1018–1038 (1975).

[b6] Baibich M. N. . Giant magnetoresistance of (001)Fe/(001) Cr magnetic superlattices. Phys. Rev. Lett. 61, 2472–2475 (1988). 10039127 10.1103/PhysRevLett.61.2472

[b7] Parkin S. S. P. . Giant tunnelling magnetoresistance at room temperature with MgO (100) tunnel barriers. Nature Matter. 3, 862–867 (2004). 10.1038/nmat1256 15516928

[b8] Jin S. . Thousandfold change in resistivity in magnetoresistive LaCaMnO films. Science 264, 413–415 (1994). 17836905 10.1126/science.264.5157.413

[b9] Xu R. . Large magnetoresistance in non-magnetic silver chalcogenides. Nature 390, 57–60 (1997).

[b10] Hu J. S., Rosenbaum T. F. & Betts J. B. Current jets, disorder, and linear magnetoresistance in the silver chalcogenides. Phys. Rev. Lett. 95, 186603 (2005). 16383932 10.1103/PhysRevLett.95.186603

[b11] Solin S. A., Thio T., Hines D. R. & Heremans J. J. Enhanced room-temperature geometric magnetoresistance in inhomogeneous narrow-gap semiconductors. Science 289, 1530–1532 (2000). 10968784 10.1126/science.289.5484.1530

[b12] Sinchenko A. A., Latyshev Y. I., Orlov A. P. & Monceau P. Anomalous asymmetry of magnetoresistance in NbSe_3_ single crystals. Jetp Lett. 84, 271–274 (2006).

[b13] Petrovic C. . Anisotropy and large magnetoresistance in the narrow-gap semiconductor FeSb_2_. Phys. Rev. B 67, 155205 (2003).

[b14] Sun Z. G., Mizuguchi M., Manago T. & Akinaga H. Magnetic-field-controllable avalanche breakdown and giant magnetoresistive effects in Gold semi-insulating-GaAs Schottky diode. Appl. Phys. Lett. 85, 5643–5645 (2004).

[b15] Chen J. J. . Large positive magnetoresistance in germanium J. Appl. Phys. 11, 114511 (2014).

[b16] Schoonus J. J. H. M., Bloom F. L., Wagemans W., Swagten H. J. M. & Koopmans B. Extremely large magnetoresistance in boron-doped silicon. Phys. Rev. Lett. 100, 127202 (2008). 18517905 10.1103/PhysRevLett.100.127202

[b17] Delmo M. P., Yamamoto S., Kasai S., Ono T. & Kobayashi K. Large positive magnetoresistive effect in silicon induced by the space-charge effect. Nature 457, 1112–1116 (2009). 19242471 10.1038/nature07711

[b18] Porter N. A. & Marrows C. H. Linear magnetoresistance in n-type silicon due to doping density fluctuations. Sci. Rep. 2, 565 (2012). 22876340 10.1038/srep00565 PMC3413879

[b19] Wan C. H. . Nonlocal magnetoresistance due to Lorentz force in linear transport region in bulk silicon. Appl. Phys. Lett. 103, 262406 (2013).

[b20] Schoonus J. J. H. M., Haazen P. P. J., Swagten H. J. M. & Koopmans B. Unravelling the mechanism of large room-temperature magnetoresistance in silicon. J. Phys. D. Appl. Phys. 42, 185011 (2009).

[b21] Porter N. A. & Marrows C. H. Dependence of magnetoresistance on dopant density in phosphorous doped silicon. J. Appl. Phys. 109, 07C703 (2011).

[b22] Delmo M. P., Shikoh E., Shinjo T. & Shiraishi M. Bipolar-driven large linear magnetoresistance in silicon at low magnetic fields. Phys. Rev. B 87, 245301 (2013).

[b23] Wan C., Zhang X., Gao X., Wang J. & Tan X. Geometrical enhancement of low-field magnetoresistance in silicon. Nature 477, 304–308 (2011). 21921912 10.1038/nature10375

[b24] Parish M. M. & Littlewood P. B. Non-saturating magnetoresistance in heavily disordered semiconductors. Nature 426, 162–165 (2003). 14614501 10.1038/nature02073

[b25] Guttal V. & Stroud D. Model for a macroscopically disordered conductor with an exactly linear high-field magnetoresistance. Phys. Rev. B 71, 201304 (2005).

[b26] Parish M. M. & Littlewood P. B. Classical magnetotransport of inhomogeneous conductors. Phys. Rev. B 72, 094417 (2005).

[b27] Stroud D. & Bergman D. J. New exact results for the Hall coefficient and magnetoresistance of inhomogeneous two-dimensional metals. Phys. Rev. B 30, 447–449 (1984).

[b28] Parish M. M. Magnetocapacitance without magnetism. Phil. Trans. R. Soc. A 372, 20120452 (2014). 24421378 10.1098/rsta.2012.0452 PMC3895979

[b29] Abrikosov A. A. Quantum magnetoresistance. Phys. Rev. B 58, 2788–2794 (1998).

[b30] Abrikosov A. A. Quantum linear magnetoresistance. Europhys. Lett. 49, 789–793 (2000).

[b31] Yang D. Z. . A Large magnetoresistance effect in p-n junction devices by the space-charge Effect. Adv. Funct. Mater. 23, 2918–2923 (2013).

[b32] Wang T. . Angular dependence of the magnetoresistance effect in a silicon based p-n junction device. Nanoscale 6, 3978–3983 (2014). 24561960 10.1039/c3nr04077a

[b33] Devegvar P. G. N., Levy L. P. & Fulton T. A. Conductance fluctuations of mesoscopic spin-galsses. Phys. Rev. Lett. 66, 2380–2383 (1991). 10043470 10.1103/PhysRevLett.66.2380

[b34] Jaroszynski J., Wrobel J., Karczewski G., Wojtowicz T. & Dietl T. Magnetoconductance noise and irreversibilities in submicron wires of spin-glass n(+)-Cd_1-x_Mn_x_Te. Phys. Rev. Lett. 80, 5635–5638 (1998).

[b35] Cheng X. M. . Antisymmetric magnetoresistance in magnetic multilayers with perpendicular anisotropy. Phys. Rev. Lett. 94, 017203 (2005). 15698126 10.1103/PhysRevLett.94.017203

[b36] Segal A., Shaya O., Karpovski M. & Gerber A. Asymmetric field dependence of magnetoresistance in magnetic films. Phys. Rev. B 79, 144434 (2009).

