# Supplementary material for: Temperature-Dependent Asymmetry of Anisotropic Magnetoresistance in Silicon p-n Junctions
Source: Sci Rep. 2015 Sep 1;5:11096. doi: 10.1038/srep11096 (PMC4555100; doi:10.1038/srep11096)
Supplement: Supplementary Information [file srep11096-s1.pdf]

Temperature-Dependent Asymmetry of Anisotropic Magnetoresistance in Silicon *p-n* Junctions

D. Z. Yang,[1] T. Wang,[1] W. B. Sui,[1] M. S. Si,[*1] D. W. Guo,[1] Z. Shi,[2] F. C. Wang,[1] and D. S. Xue[1*]

[1]*Key Laboratory for Magnetism and Magnetic Materials of Ministry of Education, Lanzhou University, Lanzhou 730000, China.*

[2]*The Department of physics, Tongji University, Shanghai 200092, China*

Contents

In Fig S-1，one can easily find that without the magnetic field the resistance of junction is weakly temperature-dependent. However, at H =2.0 T, the resistance of junction sharply increases with the decreasing temperature. This is because that the space charge region is more efficiently affected by the magnetic field due to the enhancement of the carrier mobility at lower temperature.

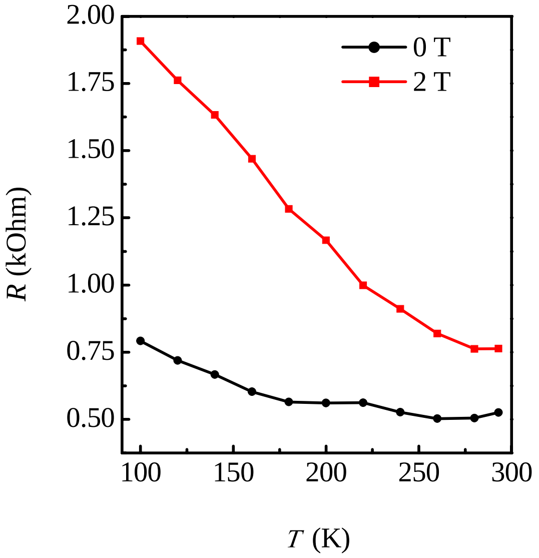

Fig. S-1. The resistance of junction at 20 mA as a function of temperature with magnetic fields 0 T and 2 T. The lines are guided to eye.

* E-mail: sims@lzu.edu.cn
* E-mail: xueds@lzu.edu.cn
